# Addressing critical needs in the fight to end tuberculosis with innovative tools and strategies

**DOI:** 10.1371/journal.pmed.1002795

**Published:** 2019-04-30

**Authors:** Mark Hatherill, Richard E. Chaisson, Claudia M. Denkinger

**Affiliations:** 1 South African Tuberculosis Vaccine Initiative (SATVI), University of Cape Town, Cape Town, South Africa; 2 Center for Tuberculosis Research, Johns Hopkins University School of Medicine, Baltimore, Maryland, United States of America; 3 Center of Infectious Diseases, University of Heidelberg, Heidelberg, Germany; 4 FIND, Geneva, Switzerland

## Abstract

This month in PLOS Medicine we launched a Special Issue on New Tools and Strategies for Tuberculosis Diagnosis, Care, and Elimination. In this issue’s Editorial, the Guest Editors Claudia Denkinger, Richard Chaisson, and Mark Hatherill highlight some of the research that will publish and how these studies focusing on discovery, clinical trials and implementation research collectively add to the prospects for reaching the EndTB targets of the WHO by 2035.

More than 130 years after Koch first described the tubercle bacillus and its etiologic role in tuberculosis (TB) to the Physiologic Society of Berlin, TB is still responsible for an appalling human toll and is the leading single infectious cause of death worldwide [1]. In September 2018, the United Nations General Assembly held a High-Level Meeting on Tuberculosis during which multiple heads of state and ministers of health pledged to end the global TB epidemic by 2035, setting ambitious targets for diagnosing and treating cases, providing preventive therapy to those most at risk, and making investments in TB care and research [2]. It is clear that the modest decline in global TB incidence and mortality in recent years needs to be accelerated to reach the “End TB” targets for 2035 [3]. This can only be achieved if the implementation of current approaches to TB control is optimized and new tools and strategies for TB prevention, screening, diagnosis, and treatment are developed. This special issue highlights the breadth of the approaches currently available, promising new developments as well as identifying some of the challenges still ahead.

The potential for impact of new strategies is not limited to biomedical intervention but includes societal and socioeconomic approaches to change the paradigm of TB case finding and treatment. In this issue, Rubinstein and colleagues evaluate conditional cash transfer as part of a social support policy to promote adherence to improve treatment outcomes for the most vulnerable TB patients [4]. Other approaches with potential to make substantial progress include strategies to engage private healthcare providers to find the missing millions of cases and efficiently refer patients to receive timely and appropriate treatment.

This issue also takes a new look at old tools. In a pragmatic cluster-randomized trial, Hanrahan and colleagues showed that contact tracing, based on symptom screening and Xpert MTB/RIF testing (Cepheid, Sunnyvale, CA), did not increase the rate of TB treatment initiation compared to facility-based screening [5]. These findings highlight the need for more sensitive triage and diagnostic tests and raise questions about the role of symptoms as the principal entry point for TB screening in high-incidence countries of sub-Saharan Africa, given the success of a contact tracing strategy incorporating both symptom screening and chest radiography in Vietnam [6].

Host markers have been targeted most widely either with proteomic or transcriptomic approaches in the pursuit of a triage test that might even identify patients at the stage of incipient TB, i.e., in an asymptomatic phase with early disease. In this issue, Khatri and colleagues compared 16 transcriptional signatures on 24 data sets that span the geographic regions of the global epidemic [7]. Although the work confirms the promise of a three-gene transcript signature in particular [8], the feasibility of translation into an affordable, easy-to-use triage test for use in high-burden developing countries remains elusive, given the need for multiplexing and quantitation, based on costs of currently commercialized PCR chemistry and integrated instrumentation. An application as an incipient TB test for high-income countries, however, is becoming more and more realistic. A proteomic approach is more likely to be translated into an affordable triage test, provided that 3 or fewer markers are targeted. However, all published proteomic signatures, including the ones pursued by Scriba and colleagues, require broader validation considering different co-infections, including but not limited to HIV [9].

When it comes to non-sputum biomarker tests for stand-alone diagnosis, recent publications have demonstrated the potential of Lipoarabinomannan (LAM) for sensitive detection of active TB [10]. The potential impact of these novel, more sensitive tests in the pipeline can be discerned from the work by Huerga and colleagues in this issue showing that the only currently available commercial test targeting LAM, even at highly suboptimal sensitivity, can have a significant impact even outside of patient groups currently recommended by WHO [11]. The value of LAM as a marker for treatment monitoring in sputum is highlighted by Liu and colleagues [12]. Given that both phenotypic and genotypic drug susceptibility testing (DST) is limited for drugs newly recommended for drug-resistant TB (e.g., bedaquiline), improved treatment monitoring tools are becoming even more important.

Advances in treatment of TB in recent years have been limited to multidrug-resistant (MDR) and extensively drug-resistant (XDR) TB [13,14], but the treatment of drug-resistant TB still involves regimens with considerable toxicity and shortening the treatment of drug-susceptible TB has been difficult [15]. In this special issue, Savic and colleagues report on meticulous pharmacokinetic and pharmacodynamics evaluations of drug penetration into pulmonary lesions in patients undergoing lung resection, providing important insights into how a number of currently available agents reach or don’t reach the site of disease [16]. These observations will be useful in designing and evaluating new regimens, as well as understanding the potential of new agents as they undergo preclinical evaluation.

The limitations of our therapeutic armamentarium are apparent in the unacceptable mortality rate among HIV co-infected patients hospitalized with TB. Gupta-Wright and colleagues describe the development and validation of a simple clinical score, including urine LAM, to identify co-infected patients at higher risk of death both pre- and post-discharge [17]. Yet, even if we could identify these high-risk patients, changing patient outcomes with early and more effective interventions remains a major challenge.

Our therapeutic limitations spotlight the critical need for more effective TB prevention. Recent trials show that shorter, simplified preventive drug regimens are possible [18]. In a Perspective, Churchyard and Swindells discuss the importance of strategies to target latent TB infection in high-risk populations and thus disrupt a reservoir for new infections in high-burden countries [19]. In another Perspective, Vekemans and colleagues discuss some of the recent breakthroughs in the search for an effective TB vaccine [20]. These include positive results from 2 vaccine trials of the subunit vaccine M72/AS01_E_ and of Bacille Calmette Guerin (BCG) revaccination, which support empirical efficacy testing both for novel vaccine candidates and for existing vaccines using new approaches [21,22]. One such novel approach is aerosol vaccine delivery to the lung—the site of infection. In this issue, McShane and colleagues describe an experimental medicine trial to explore alternating aerosol and intradermal vaccination routes to boost the immune response to the *Mycobacterium tuberculosis* antigen 85A [23]. The results highlight the importance of understanding mucosal and systemic immune responses to aerosolized candidate vaccines and the need to optimize tolerability of such vaccination regimens.

Collectively, this special issue highlights the vibrant field of discovery, clinical trials, and implementation research that will enable the End TB targets of the World Health Organization. While there is much promise in new diagnostics, biosignatures, drugs, vaccines, and innovative implementation strategies, this special issue also highlights that, although we have come a long way from Koch, there is a long path still to be walked in order to bend the curve on this ancient disease.

## Abbreviations

BCG: Bacille Calmette Guerin
DST: drug susceptibility testing
LAM: Lipoarabinomannan
MDR: multidrug-resistant
TB: tuberculosis
XDR: extensively drug-resistant
